# Magnetoreception System in Honeybees (*Apis mellifera*)

**DOI:** 10.1371/journal.pone.0000395

**Published:** 2007-04-25

**Authors:** Chin-Yuan Hsu, Fu-Yao Ko, Chia-Wei Li, Kuni Fann, Juh-Tzeng Lue

**Affiliations:** 1 Department of Life Science, Chang Gung University, Tao-Yuan, Taiwan; 2 Department of Life Science, National Tsing Hua University, Hsinchu, Taiwan; 3 Department of Philosophy, Atkinson College, York University, Toronto, Canada; 4 Department of Physics, National Tsing Hua University, Hsinchu, Taiwan; Centre de Recherches su la Cognition Animale-Centre National de la Recherche Scientifique and Université Paul Sabatier, France

## Abstract

Honeybees (*Apis mellifera*) undergo iron biomineralization, providing the basis for magnetoreception. We showed earlier the presence of superparamagnetic magnetite in iron granules formed in honeybees, and subscribed to the notion that external magnetic fields may cause expansion or contraction of the superparamagnetic particles in an orientation-specific manner, relaying the signal via cytoskeleton (Hsu and Li 1994). In this study, we established a size-density purification procedure, with which quantitative amount of iron granules was obtained from honey bee trophocytes and characterized; the density of iron granules was determined to be 1.25 g/cm^3^. While we confirmed the presence of superparamagnetic magnetite in the iron granules, we observed changes in the size of the magnetic granules in the trophycytes upon applying additional magnetic field to the cells. A concomitant release of calcium ion was observed by confocal microscope. This size fluctuation triggered the increase of intracellular Ca^+2^ , which was inhibited by colchicines and latrunculin B, known to be blockers for microtubule and microfilament syntheses, respectively. The associated cytoskeleton may thus relay the magnetosignal, initiating a neural response. A model for the mechanism of magnetoreception in honeybees is proposed, which may be applicable to most, if not all, magnetotactic organisms.

## Introduction

The waggle dances of honeybees communicate the information of direction and distance from the hive to a new food source was first hypothesized by von Frisch [1]. Subsequent studies show that the information of distance depends on optic flow (retinal image flow) perceived in the flight to the food source and is conveyed by the duration of the waggle dance [2]–[4]. The information of direction, on the other hand, depends on the angle between the heading to the food source and the sun azimuth and is conveyed by the angle between the waggling segment of the dance and the vertical [5]. Bees use different compass for navigating in a complex environment. Besides the azimuthal position of the sun [5], a polarized light compass is also employed [6]–[7] and landmarks may also be useful when days are cloudy [8]. Other sensory cues have been suggested as orientation reference in the framework of bee navigation. Among them, the information provided by the magnetic field has been repeatedly invoked to account for bee orientation performances [9]–[22].

Magnetoreception of honeybees has been proposed on the basis of numerous behavioral evidences. They can be summarized as follows: The behavioral changes in comb building, and homing orientation when extra magnetic field is added [10]–[12], [17], [19], [20]. Tiny magnets glued to the honeybee abdomen near the region of known magnetite concentration in the anterior dorsal abdomen interfere with magnetic discrimination in choice experiments [21]. Free-flying honeybees are able to detect static intensity fluctuations as weak as 26 nT against the earth-strength magnetic field [22]. Honeybees' behavior in T-maze experiments is affected by a brief magnetic pulse [17], which is a unique ferromagnetic effect. Free-flying honeybees can be trained in discrimination experiments to respond to local magnetic anomalies [15], [17], [21]–[23]. Free-flying honeybees are able to detect alternating fields (430µT) at frequencies of 10 and 60 Hz [18]. These evidences suggest that biomagnetites (Fe_3_O_4_) may be present as magnetoreceptor, which plays a crucial role as a transducer of the magnetic field information.

To explain the behavior of magnetoreception and to search for biomagnetites, researches on magnetic remanence and iron biomineralization in honeybees have been carried out for a long time. The results of these works can be summarized as follows: The magnetic remanence was detected in the abdomen of honeybees by superconducting quantum-interference device magnetometers (SQUID) [9]. Iron granules (IGs), 0.5±0.1 µm in diameter, were found in the trophocytes that encircle the abdomen under cuticle and are located mainly at the ventral abdomen [24]–[27]. They are randomly distributed in the cytoplasm of trophocytes. The formation processes of IGs have also been elucidated. IGs were intracellularly deposited in the iron deposition vesicles (IDVs) of trophocytes and cytoskeletons were attached to the membrane of IDVs [13], [27]. Elemental composition analysis indicates that IGs are mainly comprised of iron, phosphorus and minor amounts of calcium [24]–[27]. Superparamagnetic magnetite (SM) was observed in four IGs of trophocyte under high-resolution transmission electron microscopy (HRTEM) [13]. Typical X-band resonance spectra were obtained from the abdomen of honeybee by electron paramagnetic resonance (EPR) and showed that Fe_3_O_4_ and FeOOH are present in the abdomen of honeybee [28]. A hysteresis curve was obtained from the whole body and abdomen of honeybee by SQUID and showed that intrinsic coercivity is at the interval of 83-103 Oe and 44 Oe, respectively [29], [30]. These results suggest that honeybees have biomagnetites, which, in addition to behavioral evidences, indicates that they have a capacity for magnetoreception.

All of magnetic remanence from SQUID, the typical X-band resonance spectra from EPR, and hysteresis curve from SQUID were obtained from the whole body or abdomen of honeybees. In addition, SM was observed in four IGs of trophocyte. Whether the results of magnetic measurement are derived from the SM of IGs remains an open question. Although SM was observed in the IGs of trophocyte under HRTEM, the study was carried out on a very small sample (four IGs) [13]. Further evidence is required to determine the presence of SM in IGs. The evidences of behavior and iron biomineralization suggest that honeybees have a capacity for magnetoreception. However, no cellular evidence was found to explain that capacity until now.

In this study, we establish a size-density purification procedure to recover quantitative amount of IGs from trophocytes, needed for characterization and demonstrating the presence of SM in IGs. We determined the magnetic properties of the purified IGs by SQUID, atomic force microscope (AFM), magnetic force microscope (MFM), EPR, and electron spectroscopy for chemical analysis (ESCA). We also applied additional magnetic field (1 Gauss) to examine whether the size of IGs in trophocytes can be induced to change along with release of calcium ions.

## Results

### IGs purification and characterization

**Figure 1:**
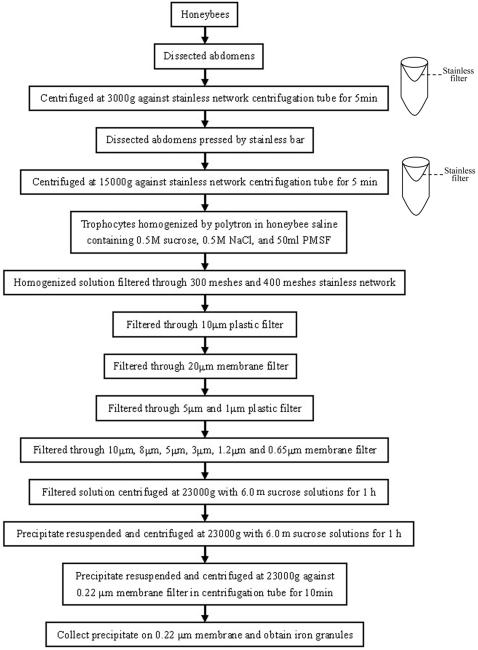
A flow diagram showing the purification processes of the size-density purification technique.

**Figure 2:**
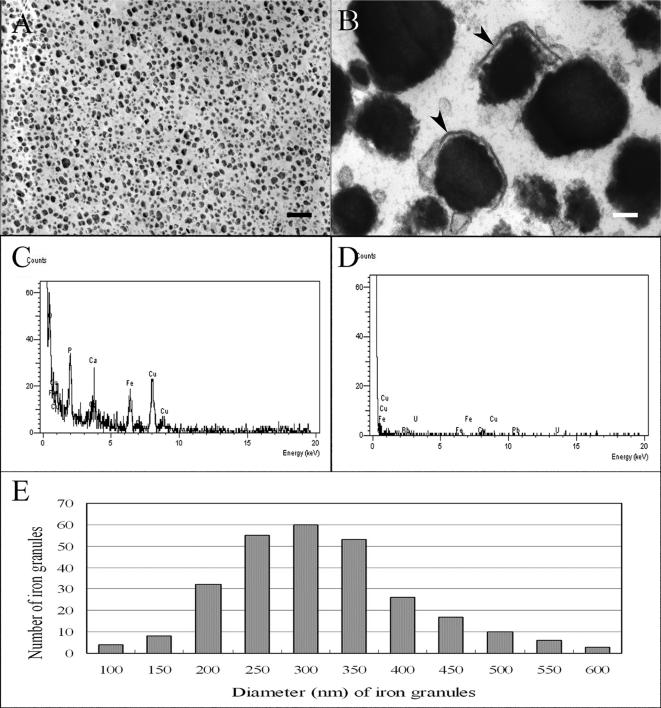
IGs purified from the trophocytes of honeybees. (A) A large amount of IGs appear in precipitates purified from the trophocytes. Scale bar, 1 µm. (B) Purified IGs enclosed with lipid-bilayer membranes (arrowhead). Scale bar, 100 nm. (C) EDX spectrum is obtained from an IG in the purified precipitates. Iron (Fe), calcium (Ca) and phosphorus (P) are present in an IG. STEM mode of 100-kV accelerating voltage was used; counts were made for 100 seconds. (D) EDX spectrum of spurr's resin is devoid of IGs in the purified precipitates. The experimental conditions are the same as in C. (E) Size distribution of purified IGs calculated from the TEM pictures (N = 274). N, total number of granules used in this calculation.

In this study, we established a size-density procedure new for purification of IGs from trophocytes (Figure 1). Numerous electron-dense granules were observed in the purified precipitates by transmission electron microscope (TEM) (Figure 2A). The morphology of these granules is similar to that of IGs in the trophocytes, but only a few retained their vesicle membrane (Figure 2B). With energy dispersive X-ray spectrum microanalyzer under scanning transmission electron microscope (STEM), these electron-dense granules is shown to contain a higher concentration of iron, phosphorus and a lower concentration of calcium as compared to spurr's resin, a control. Other elements were noted, with copper (Cu) derived from the support grid and lead (Pb) and uranium (U) from TEM stain solution (Figure 2C and 2D). The size of these electron-dense granules ranged from 0.1 µm to 0.6 µm in diameter, with an average of 0.3±0.1 µm (N = 274) (Figure 2E). Energy dispersive X-ray spectrum of individual granule is consistent with that of IGs in the trophocytes of honeybees previously reported [24]–[26].

The morphology of electron-dense granule vesicles in the purified precipitates is similar to that of IDVs observed in the trophocytes of adult workers [24]–[26]. These results suggest that electron-dense granules in the purified precipitates are IGs of the trophocytes.

The density of the purified IGs was measured. This was done by first precipitating the granules by centrifugation against 6.0 molality (m) sucrose solutions at 23,000×g, with the precipitates re-suspended on 6.25 m sucrose solutions. The density of 6.0 m and 6.25 m sucrose solution is 1.2506 g/cm^3^ and 1.2539 g/cm^3^, respectively. Therefore, the density of IGs should be at the interval of 1.2506–1.2539 g/cm^3^.

### SM in IGs

Various magnetic properties of purified IGs were determined.

**Figure 3:**
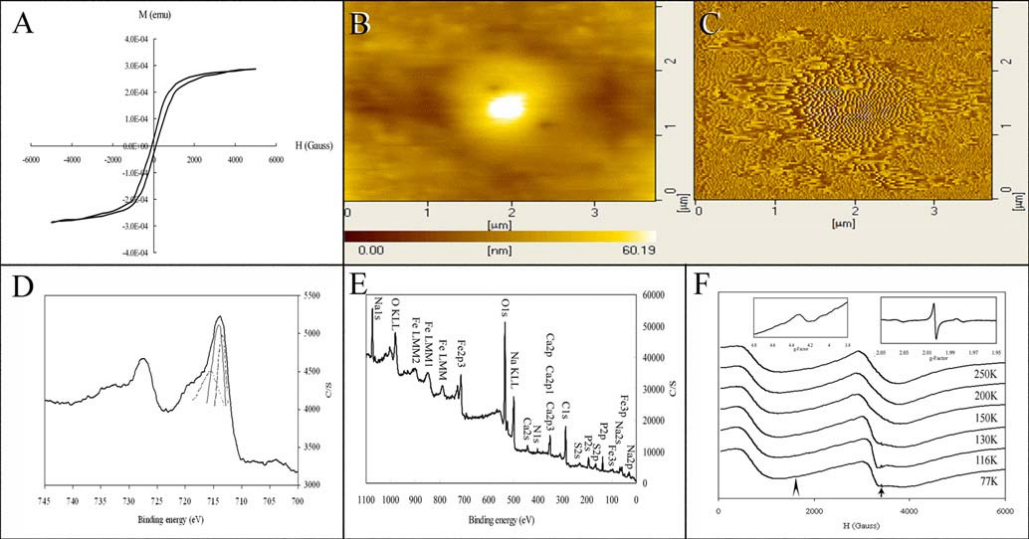
The magnetic properties of IGs determined by magnetic-determining techniques. (A) An averaged magnetization curve of SQUID from the purified IGs at 300°K for six runs. (B) An image of AFM from one of the purified IGs. (C) An image of MFM from one of the purified IGs. (D) A spectrum of ESCA from the purified IGs. The left thin line shows the Fe(2p1/2) of FeOOH. The middle thin line shows the Fe(2p3/2) of Fe2O3. The right thin line shows the Fe(2p1/2) of FeO. (E) A photoelectron spectrum of elemental composition of ESCA from the purified IGs. (F) Typical X-band resonance spectra from the purified IGs at different temperatures. Right inset shows a magnified image from the arrow. Left inset shows a magnified image from the arrowhead.

By the use of SQUID magnetometers, an averaged SQUID magnetization curve showed that IGs possess a residual magnetization of 2.5×10^−5^ emu, a saturation magnetization of 2.83×10^−4^ emu, and an intrinsic coercivity of 100–150 Gauss (Figure 3A). Hence, these IGs are magnetically soft. The soft-hysteresis loops reflect the easy axis of magnetization, which is in-plane on the surface, plausibly as a result of the magnetite granules being formed in the trophocytes. The smallness of coercivity confirms that the magnetite exists in a superparamagnetic state.

Imaging by AFM and MFM of an IG showed that single domain inherent in each IG reflects superparamagnetic behavior (Figure 3B and 3C). Ripples appeared tightly when the MFM tip scanned across a strong domain walls, as the scan speed was accelerated and became direction dependent. The AFM and MFM experiments were run in dynamic, non-contact mode under high vacuum conditions to enhance the sensitivity.

EPR spectra also show that SM is present in IGs (Figure 3F). The much sharper, yet weak, signal in the inset occurring at g∼2.0 is the spin resonance of Fe^2+^ ions from SM. The extremely small peak centered at g∼4.3 is the isolated spin ^6^S_5/2_ of the remnant Fe^3+^ ions, which are present in ferritin and FeOOH, as the second–order crystal field coefficient with axial symmetry vanishes while that with rhombic symmetry persists. Our spectroscopic results with the magnetite indicate that IGs apparently contain single-domain SM, supportive of previous studies by HRTEM [13].

The ESCA spectra of IGs showed that FeO and Fe_2_O_3_ coexist in IGs (Figure 3D). The binding energy of Fe(2p_1/2_) of FeOOH shifted about 715.6 eV and that of the Fe(2p_1/2_) of FeO about 713.2 eV, while carbon (1s) shifted about 3.8 eV (data not shown). The shifts of Fe(2p_3/2_) and Fe(2p_1/2_) of Fe_2_O_3_ were about 713.8 eV and 727.5 eV, respectively. The results show that the IGs contain Fe_3_O_4_ and FeOOH. Additionally, the spectrum of ESCA indicates that the purified IGs contained oxygen, carbon, nitrogen, iron, phosphorus, and calcium (Figure 3E). It showed little if any contamination in a large amount of purified IGs. Our spectroscopic observation is consistent with that obtained with energy dispersive X-ray spectrum microanalyzer, which showed no other contaminations.

These results demonstrate unequivocally that SM is present in the purified IGs, and IGs are magnetic granules (MGs).

### Additional magnetic fields (1 Gauss) induces the fluctuation of MGs in size

**Figure 4:**
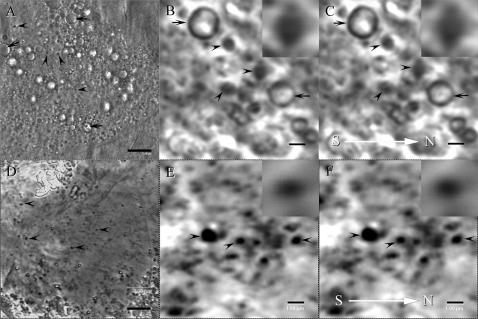
The images of MGs in the trophocytes obtained by confocal microscope. (A) MGs (arrowhead) appear as tiny black particles under low magnification in the live trophocytes. Arrow indicates oil body. Scale bar, 8 µm. (B) MGs (arrowhead) appear as black granules under high magnification in the live trophocytes. The images of MGs are obtained without the application of 1 Gauss magnetic field. Arrow indicates oil body. Inset shows a magnified MG. Scale bar, 1 µm. (C) The same images obtained by the application of 1 Gauss magnetic field in the live trophocytes. White arrow indicates the direction of magnetic field. Arrowhead indicates MGs. Arrow indicates oil body. Inset shows a magnified MG. Scale bar, 1 µm. (D) MGs (arrowhead) appear as tiny black particles under low magnification in the dead trophocytes. Scale bar, 8 µm. (E) MGs (arrowhead) appear as black granules under high magnification in the dead trophocytes. The images of MGs are obtained without the application of 1 Gauss magnetic field. Inset shows a magnified MG. Scale bar, 1 µm. (F) The same images obtained by the application of 1 Gauss magnetic field in the dead trophocytes. Arrowhead indicates MGs. White arrow indicates the direction of magnetic field. Inset shows a magnified MG. Scale bar, 1 µm.

To study the role of MGs as magnetoreceptors, we examined under confocal microscope size fluctuation of MGs in trophocytea when additional magnetic field (1 Gauss) was applied. At low magnification, MGs appear as tiny black particles in both live (Figure 4A) and dead trophocytes (Figure 4D). At high magnification, MGs appear as black granules, in live (Figure 4B) and dead trophocytes (Figure 4E). After applying 1 Gauss magnetic field for 2 min, MGs in live trophocytes shrank 12±1.7% (N = 20) at the paralleled direction to magnetic field and enlarged 1.2±0.5% (N = 20) at the vertical direction in the horizontal plane (Figure 4C). In dead trophocytes, they reduced 13±1.4% (N = 20) in size at the paralleled direction to magnetic field and enlarged 4.4±0.9% (N = 20) at the vertical direction in the horizontal plane (Figure 4F). The results show that additional magnetic field can induce the size changes in MGs, owing to MGs' magnetoreceptor property.

### Additional magnetic fields (1 Gauss) induces the release of calcium ions from trophocytes, which can be inhibited by colchicines and latrunculin B

**Figure 5:**
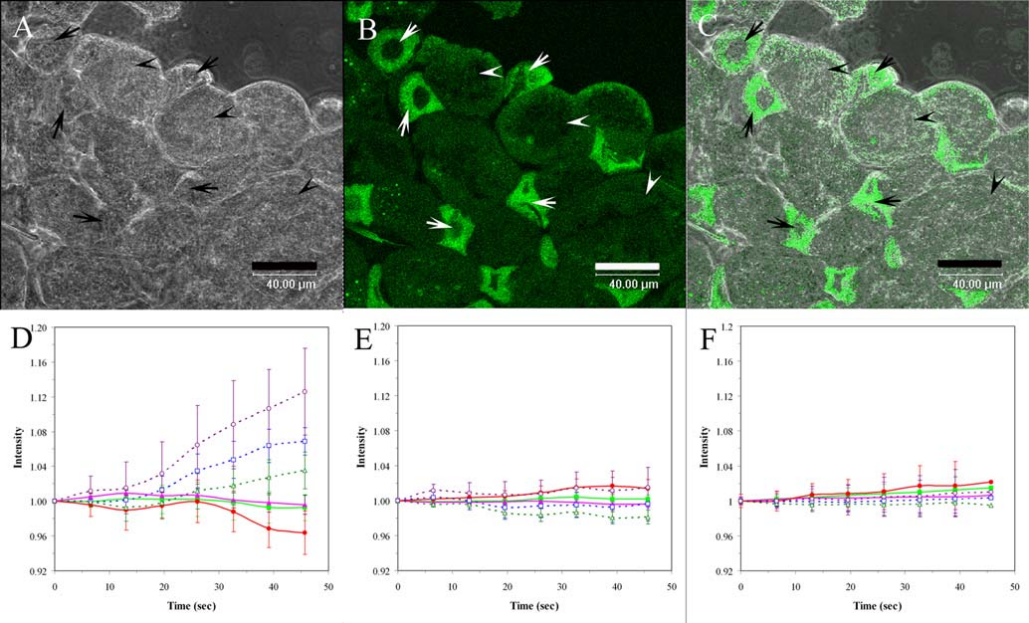
The images of increase of [Ca^+2^]i in the live trophocytes obtained by confocal microscope. (A) The image of trophocytes and fat cells under confocal microscope. Trophocytes (arrowhead). Fat cells (arrow). Scale bar, 40 µm. (B) The image of trophocytes and fat cells labeled fluo 4 under confocal microscope. Trophocytes (arrowhead). Fat cells (arrow). Scale bar, 40 µm. (C) The merge of Figure A and Figure B. Scale bar, 40 µm. (D) The fluorescence intensity of trophocytes (○), fat cells (Δ), and trophocytes and fat cells together (□) with 1 Gauss magnetic field. The fluorescence intensity of trophocytes (•), fat cells (▴) and trophocytes and fat cells together (▪) without 1 Gauss magnetic field as control. (E) The fluorescence intensity of trophocytes (○), fat cells (Δ), and trophocytes and fat cells together (□), which is inhibited by colchicine and with 1 Gauss magnetic field. The fluorescence intensity of trophocytes (•), fat cells (▴) and trophocytes and fat cells together (▪), which is inhibited by colchicine without 1 Gauss magnetic field as control. (F) The fluorescence intensity of trophocytes (○), fat cells (Δ), and trophocytes and fat cells together (□), which is inhibited by latrunculin B and with 1 Gauss magnetic field. The fluorescence intensity of trophocytes (•), fat cells (▴) and trophocytes and fat cells together (▪), which is inhibited by latrunculin B without 1 Gauss magnetic field as control.

To study signal transduction elicited by MGs for magnetoreception, the variation of calcium ion in trophocytes and fat cells labeled with flou 4 were measured under confocal microscope. Of these two types of cells, trophocytes are larger (about 75 µm in diameter) and are dark green in color, while fat cells smaller (about 30 µm in diameter) and bright green (Figure 5A and 5B), distinguishable even mixed (Figure 5C). Cells without magnetic field for about 46 seconds were used as a control, whose intracellular Ca^+2^ concentrations ( [Ca^+2^]i ) would remain constant (Figure 5D). [Ca^+2^]i was allowed to stay constant for about 12 sec. before the magnetic field was applied; the increases in [Ca^+2^]i lasted for about 34 sec (Figure 5D). By contrast, the increase of [Ca^+2^]i occur principally in trophocytes, only weakly in the fat cells.

The effect of magnetic field on [Ca^+2^]i could be inhibited by colchicine (Figure 5E) and latrunculin B (Figure 5F), which block the syntheses of microtubule and microfilament, respectively.

Taken together, these results show that the increase of [Ca^+2^]i in trophocytes may be caused by the fluctuation of MGs in size and is mediated by microtubules and microfilaments.

## Discussion

In this study, we established a new procedure based on size-density for the purification of large quantities of IGs from honeybee trophocytes. With this procedure, we verified that SM was present in IGs. We determined the density of IG to be 1.25 g/cm^3^. We showed that additional magnetic field (1 Gauss) induces size fluctuation in MGs, which triggers the increase of [Ca^+2^]i via cytoskeletons. A cellular mechanism which allows honeybees to have such a sensitive system for orientation and positioning is thus formulated.

### Physico-Chemical properties of IGs

It has been reported that IGs occupy approximately 7% of the volume of the trophocytes in honeybees [24]. We estimate that our purified IGs consisted of approximately 84% of the preparation, indicating a 12 fold purification. It is interesting to note that the purified IGs did not condensate even under a centrifugal force of 23,000×g; instead they were loosely packed, maintaining a short distance (about 50±10 nm) between each granules. In magnetotactic bacteria, there are organic materials tightly interconnected with individual particles in the cluster of magnetosome [31], [32]. Removal of the membrane by sodium dodecysulfate caused their agglomeration [31]. We observed that most of the vesicle membranes were lost. The loose packing feature of IGs may be explained by their weak magnetic force.

Phosphorus is present in IGs of bacteria and honeybees [24]–[27], [33], [34]. We surmise that phosphorus in honeybees may function as energy sources for ATP synthesis, while in bacteria they may carry additional functions such as a regulator for stress and survival, and a chelator of toxic metal ions [34]–[36]. Phosphorus in honeybees does not seem to function as a chelator of metal ions for reducing their toxicity, because pollen contains numerous elements [37] and IGs only chelate iron elements and calcium elements. In addition, 7.5 nm particles are the basic building blocks and tightly packed into core granules in the center of IDVs during the formation of IGs [27] and energy would be needed to move the 7.5 nm particles to the center of IDVs. The density value of IGs is 1.25 g/cm^3^, and that of Fe3O4 is 5.24 g/cm^3^. If the volume of an IG with a diameter of 0.6 µm is 1.13×10^−13^ ml and the volume of SM in an IG is 4.4×10^−15^ ml [14], the density value of IGs without SM would be 1.09 g/cm^3^. The actual density value of IGs is much lower than the estimated values from previous reports [24], [38].

### IGs possess SM and are MGs

It has been suggested that the IGs found in honeybees may be related to the exclusion of biological waste or the reduction of biological toxicity of metals. However, the diet of honeybees is honey and pollen. Pollens are the main source of minerals, which include mostly a metal mineral (Ca) and a little trace elements (Al, Cr, and Fe) [37]. The iron content of pollen is averaged at 0.16±0.02 µg/mg [27]. If IGs are a part of the biological waste they should have a large amount of calcium and a small amount of iron. But, on the contrary, IGs specifically deposit a large amount of iron, phosphorus and a small amount of calcium [24]–[27]. Therefore, the accumulation of iron minerals in IGs is involved in neither the exclusion of biological wastes nor the reduction of biological toxicity of metals. More likely, its biological function is to enable IGs to serve as magnetoreceptors for magnetoreception.

Previous histological studies show that trophocytes are the only cell containing IGs in the abdomen including both the dorsal and ventral regions [24], [27] and that SM is present in IGs [13]. Previous studies with magnetism measurement showed that the abdomen of honeybees possesses magnetic remanence [9], [30] and magnetite nanoparticles [28], and that SM is present. Even though other magnetite granules may be present in places other than the abdomen [29], the magnetic remanences of the whole body of honeybees may well be partially derived from that of the abdomen, which should be from the SM in IGs.

The saturation magnetization of purified IGs is about 2.83×10^−4^ emu from the abdomen of 2000 honeybees. Thus, the saturation magnetization of purified IGs per individual is about 1.4×10^−7^ emu, which is lower than the value of 2.4×10^−7^ emu as previously reported [30]. The discrepancy may be resulted from our exclusion of IGs outside of the size range of 0.45–0.22 µm in diameter in our measurements.

Earlier studies analyzed the whole body and abdomen of honeybee by SQUID and showed that intrinsic coercivity is at the interval of 83–103 Oe and 44 Oe, respectively, as derived from hysteresis curves [29], [30]. Our study showed a higher intrinsic coercivity of purified IGs between 100–150 Gauss (Oe). While these values indicate the presence of SM and larger magnetic nanoparticle purified in IGs, the latter may be formed in vitro through extraction, as has been shown in IG purification from ants (*P. marginata*) [39].

AFM and MFM have been generally used to study SM particles [40], and to determine magnetite in rainbow trout [41]. Our study extends the use of AFM and MFM, for the first time, to the detection of SM in the purified IGs of honeybees.

EPR has been used to study the magnetic material in the whole body of migratory ants [42]–[44], and stingless bees [30], as well as in the abdomen of honeybees [28]. All of the typical X-band resonance spectra show the presence of high field (HF) and low field (LF) signals. The signal of g = 4.3 at LF is indicative of the presence of the Fe^+3^ ions, which may be from ferritin and FeOOH. Its signal intensity decreases sharply with increasing temperatures. The signal of g = 2.0 at HF is indicative of the presence of magnetic nanoparticles. Its signal intensity decreases also sharply with increasing temperatures. The typical X-band resonance spectra of purified IGs from honeybees observed in our study are similar to those reported previously [28], [30], [42]–[46], showing the presence of SM in IGs. IGs are the only source of iron minerals in the trophocytes of the abdomen including both the dorsal and ventral regions [24], [27]. On these bases, we conclude that both X-band resonance spectra are derived from the SM in IGs.

**Figure 6:**
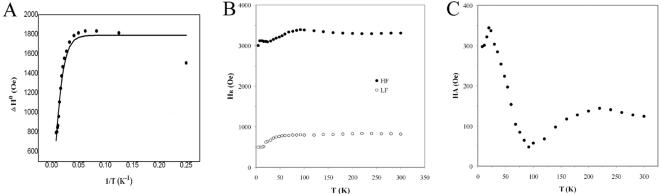
The resonance linewidth and anisotropy fields of the broad resonance lines (HF) of purified IGs. (A) Temperature dependence of the resonance linewidth of the HF. The solid line is the best fit of the HF data according to Eq. 1, with Δ*H* ^0^ = 1791±37 Oe and Δ*E*/2*k*_B_ = 50.8±2.6 K. (B) Temperature dependence of the resonance field of HF and LF. Note that the HF and LF curves are almost parallel to each other, with the field shifting by an average value of about 2496±50 Oe. (C) Anisotropy fields calculated from the resonant field's values, using Eq. 2 for the HF lines.

The temperature dependencies of the HF linewidth of purified IGs spectra are shown in Figure 6A. Based on the assumption of isolated spherical nanoparticles, the HF linewidths temperature dependencies are fitted using the equation 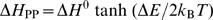
1where Δ*H* ^0^ is the low temperature limit value, Δ*E* is the magnetic energy *KV*, *V* is the nanoparticle magnetic volume, and *k*_B_ is the Boltzman constant. The solid lines in Figure 6A are the best fitting curves for Δ*H* ^0^ = 1791±37 Oe and Δ*E*/2*k*_B_ = 50.8±2.6 K, respectively. In equation 1, Δ*H* ^0^ = 5*g*β*Sn*/*D*3, the description of the prefactor Δ*H* ^0^ includes the *g* factor (*g*), the Bohr magneton (β), the spin associated with each magnetic center inside the nanoparticle (*S*), the number of magnetic centers per particle (*n*), and the particle-particle distance in the matrix (*D*). Assuming *g* = 2, *S* = 2 [45], and *n* = 8.5×10^3^ [13], we obtain *D* = 10±0.1 nm. An average shift of 2496±50 Oe between the two *H*_R_ versus *T* curves of purified IGs was obtained (Figure 6B). As *K* is temperature-dependent, Δ*E* is taken as *K*_ef_ *V* with *K*_ef_ = *Ms*(-*HA*)/2, where (*HA*) is the mean value obtained from Figure 6C (125 Oe) according to the following equation and previous studies [28], [44], [45]. 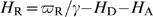
2Taking the magnetite saturation magnetization (*Ms*) as 470 Oe, magnetic volumes are estimated to be (0.48±0.02)×10^3^ nm^3^ and correspond to mean particle diameters of 9.7±0.2 nm. The size of SM in purified IGs is consistent with SM reported by previous studies of the abdomen with SQUID [30], EPR [29] and HRTEM [13]. However, larger magnetite nanoparticles (30 nm or more) were reported to be present in the abdomens of honeybees by SQUID [47]. More studies are needed to clarify the size of magnetite in the abdomen of honeybees.

The EPR spectra of de-oxygen horse spleen ferritin show that g = 9 signal can be shifted to g = 4.3 with the increment of temperature (80K to 290K) and the intensity of g = 4.3 increases with the increment of temperature (142K to 290K). g = 2.066 signal can be shifted to g = 2.014 with the increment of temperature (19K to 290K) [48]. However, the EPR spectra of purified IGs show that g = 4.3 signal appears at 77K and doesn't shift with temperature. Its intensity increases as temperature decreases. g = 2.0 signal appears at 77K and doesn't shift with temperature. Its intensity increases as temperature decreases. Taken together, ferritin is clearly present in purified IGs [49], but the EPR spectra of purified IGs are not consistent with EPR spectra of ferrintin [48]. Conversely, the EPR spectra of purified IGs are similar to the EPR spectra of the abdomen of honeybees [28], ant [44] and termites [46]. Therefore, the EPR spectra of purified IGs are not from ferrintin and should be from SM.

The EDX spectra of a large amount of purified IGs of ESCA (in this article) are the same as the EDX spectra of an iron granule of STEM [24]. It showed no contaminations in a large amount of purified IGs. The EDX spectral of ESCA not only confirms the presence of SM, but also FeOOH in IGs. Although FeOOH has been detected in the whole body of honeybees [28], the FeOOH signal should be derived from IGs. Taken together, we conclude that honeybees have SM and FeOOH, which are derived from IGs.

### Additional magnetic fields (1 Gauss) induces the release of calcium ions from trophocytes and fat cells

We show in this study the release of calcium ion occurs principally in the trophocytes, but there is a small effect observed in the fat cells. This effect is due to the magnetic field acting on fat cells. Previous studies show that exposure to a static magnetic field resulted in an increased Ca^+2^ level in numerous cells, including macrophages [50], astrocytoma cells [51], [52], and chromaffine cells [53]. This increase of [Ca^+2^]i may be mediated by the change of the structure and dynamic properties of lipid membrane [54], [55], membrane potential and cell surface charge [56], protein structure [57], [58], and intramembrane proteins distribution [59]. [Ca^+2^]i is mainly increased by either stimulation of Ca^+2^ influx mediated by the Ca^+2^ channel of the cell membrane or Ca^+2^ released from intracellular Ca^+2^ stored when a static magnetic field is applied [60], [61]. Therefore, the increase of [Ca^+2^]i of fat cells may be the effect of magnetic field similar to the effect of magnetic field on other cells. The increase of [Ca^+2^]i from fat cell is slightly induced by additional magnetic field, but the increase of [Ca^+2^]i from trophocytes is largely induced by additional magnetic field. The increase of [Ca^+2^]i from fat cell is clearly different from that of trophocytes. Furthermore, magnetic field exposure may influence intracellular functions mediated by its effect on the cell membrane in association with cytoskeletal proteins [62]. Therefore, the increase of [Ca^+2^]i from trophocytes may be due to the fluctuation of MGs in size associated with cytoskeleton.

### Magnetoreception system of honeybees

Additional magnetic field can induce the fluctuation of MGs in size, which corresponds to the property of SM [63]. This property of MGs can be used as magnetoreceptor. The effect of size fluctuation of MGs in live trophocytes is lower than that of dead trophocytes, which may be due to the resistance of the membrane of IDVs. The resistance of the membrane may be the mechanism, which provides the high sensitivity of the sense system. Previous study showed that cytoskeletons are attached to the membrane of IDVs [13], [27]. Cytoskeletons may be involved in signal transduction in the cells. Therefore, the fluctuation of MGs with the resistance of the membrane of IDVs and the association of cytoskeletons constitute a fine system to sense the change in magnetic field and to trigger the signal transduction. This system is further confirmed by the fact that additional magnetic field can induce the increase of [Ca^+2^]i, which can be inhibited by colchicines and latrunculin B. Thus, MGs associated with the membrane of IDVs and cytoskeletons can be used as magnetoreceptor to sense the magnetic field.

### A model for the magnetoreception system in honey bees

**Figure 7:**
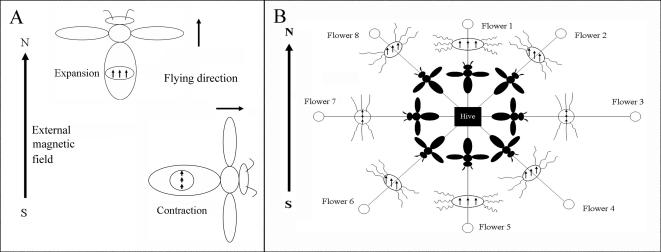
The magnetic map hypothesis of magnetoreception for orientation and positioning during foraging and homing. (A) When honeybees fly in the direction paralleled to external magnetic field, MG expands due to the repellent force of SM. When honeybees fly in the perpendicular direction to external magnetic field, MG contracts in the same direction due to the attractive force of SM. (B) The direction of magnetic field of SM is parallel to the direction of Earth's magnetic field at flower 1. SM would be in a side-by-side position, creating repulsion and subsequent expansion of the MGs. At the flower 3, the direction of magnetic field of SM is vertical to the direction of Earth's magnetic field. SM would be positioned end-to-end, creating attraction and subsequent contraction of the MGs. This contraction induces the tensing of cytoskeletons and triggers the increase of [Ca^+2^]i in trophocytes. At flower 2, the relaxing and tensing of cytoskeletons is a mixture of flower 1 and flower 3. The direction of magnetic field of SM at the flower 1 is the same as flower 5. Flower 2 is the same as flower 6, and so on. The distinction in both cases is dependent on the direction of waggle dance.

Based on the results of cellular studies, we postulate a magnetic map hypothesis for the magnetoreception as follows: the magnetic field induces the size of MGs to shrink at the paralleled direction to the magnetic field and to enlarge at the vertical direction in the horizontal plane (Figure 7A). This fluctuation in the size of MGs can induce the relaxing and tensing of cytoskeletons to trigger the increase of [Ca^+2^]i. The increase of [Ca^+2^]i can further trigger signal transduction for magnetoreception. This is probably the mechanism by which honeybees establish the magnetic map in memory during orientation flights. Thus, foragers can set course at any direction to forage and to home from any location (Figure 7B). This magnetoreception system is highly sensitive, because honeybees can detect magnetic field as low as 26 nT, compared to the 45000 nT of Earth's magnetic field [22]. In addition, each bee has about 1.87×10^11^ SM (2.2×10^8^ IGs per bee, 8.5×10^3^ SM per IG and SM occurs in 10% of IGs) [13].

According to this hypothesis, (1) The flight paths should be straight from the hive to the food source, as has been determined in the foraging and homing of honeybees [64], [65] and the migration of birds from origin to destination [66]. (2) A 360-degree magnetic map in memory would be established during orientation flights. It has been determined that orientation flights are a prerequisite for successful homing [67]–[69] and honeybees navigate according to a map-like spatial memory [65]. The magnetoreception system maybe present in pigeons and all other magnetotactic homing and migrating organisms as well. For example, pigeons released at a new site circle in the air several times to get their magnetic bearing, before finding their orientation and then fly straight home.

‘How do MGs found in the abdomen function as magnetoreceptors’ is an enigma yet to be resolved. Suffice to note that peripheral neurons of insects may play a role independent of the brain, such that a male cockroach can continue with mating, with its head bitten off by his female partner. Certainly, a magnetoreception system for positioning and orientation exists in honeybees, and this simple, primitive, and highly accurate sensing mechanism may be present in all other magnetotactic organisms.

## Materials and Methods

### Honeybees

Honeybees, *Apis mellifera*, were bred in an open environment of a bee-breeding room at the 10^th^ floor of our institute building. Sucrose and pollen grains were occasionally added to the hives as dietary supplements.

### Purification of IGs

Two thousand worker bees were freshly collected from a hive, anesthetized with ice-distilled water, washed with ice-distilled water three times, and dissected with stainless tools. Ventral abdomens were obtained, pollen washed out through stainless filter with honeybee saline (156.4 mM NaCl, 2.7 mM KCl, 1.8 mM CaCl2, 22.2 mM glucose, pH 7.3) three times, and centrifuged against a stainless network filter in a centrifugal tube at 3000×g for 2 minutes three times. The abdomens were pressed by stainless bar, dissected into small pieces, and centrifuged against a stainless network filter in a centrifugal tube at 15000×g for 5 minutes three times in honeybee saline containing 0.5 M sucrose and 0.5 M NaCl. Trophocytes were collected into 10 ml honeybee saline containing 0.5 M sucrose, 0.5 M NaCl and 50 µl phenylmethylsulfonyl fluoride, and homogenized by polytron at max speed 30,000 rpm for 3 minutes. The homogenized solution was filtered through a series of filters containing 350 meshes stainless network filter, 10 µm plastic network filter, 20 µm membrane filter, plastic network filters with 5 µm and 1 µm, as well as membrane filters with 10 µm, 8 µm, 5 µm, 3 µm, 1.2 µm, 0.8 µm, and 0.65 µm. The filtered solution was centrifuged against 6.0 m sucrose solutions at 23000×g for 1 hour. The precipitate was collected, resuspended in honeybee saline, and centrifuged against 6.0 m sucrose solutions at 23000×g for 1 hour again. The precipitate was collected, sucrose washed out with honeybee saline, and centrifuged against a 0.22 µm membrane filters in a centrifugal tube at 23000×g for 10 minutes. The precipitate on 0.22 µm membrane filters was collected and analyzed.

### TEM

The purified precipitates were fixed in 2.5% glutaraldehyde in a 0.1 M phosphate buffer with 0.35 M sucrose at pH 7.4 for 30 minutes at 25°C and were postfixed in 1% osmium tetroxide in a 0.1 M phosphate buffer with 0.35 M sucrose at pH 7.4 for 1hour at 25°C. The purified precipitates were dehydrated through an ethanol series and embedded in Spurr's resin. Thin sections (60–90 nm in thickness) were cut with a diamond knife, stained with uranyl acetate and lead citrate and then examined, using a JEOL JEM-2000EXII TEM, operating at an accelerated voltage of 100 kV. The elemental composition and crystal structure in selected areas of thin sections were analyzed by energy-dispersive X-ray microanalysis and by selected-area electron diffraction in a JEOL JEM-2000FX STEM operating at an accelerated voltage of 100 kV [27].

### SQUID magnetometer

The purified IGs from 2 thousand bees were lyophilized with a lyophilizer for six runs. The resulting powder was transferred to tubes and sealed under nitrogen flux to prevent oxygen contributions to the SQUID signal at low temperatures. The magnetization and hysteresis of each sample was measured at a temperature of 300 K and magnetic field intensity from−5000 to 5000 Gause under a SQUID magnetometer (Quantum Design MPMS2).

### AFM/MFM

The purified IGs from 8 thousand bees were prepared according to TEM techniques. Sections (100 nm in thickness) were cut with a diamond knife, and then transferred to silicon chips. A commercially available AFM/MFM (SPA300, Seiko Scientific Instrument, Tokyo, Japan) was used to generate the images. The microscope was operating in Tapping/Lift mode to obtain images of both topography and magnetic force over a region of the samples. High-resolution AFM images of the sample surface topography and magnetic interaction was provided simultaneously.

### ESCA

The purified IGs from 40 thousand bees were lyophilized with a lyophilizer. The resulting powder was pressed into a disk and characterized by ESCA on a Perkin Elmer Phi 1600 ESCA system. Each sample was analyzed by survey and high-resolution spectra employing an Al Kα X-ray anode, energy 1486.6 eV. From the survey spectra, the qualitative and quantative elemental composition and state of the uppermost layer were computed. The binding state of elements Fe was obtained by acquisition of high-resolution spectra and subsequent curve-fitting.

### EPR

The purified IGs from 80 thousand bees were lyophilized with a lyophilizer. The resulting powder was transferred to EPR quartz tubes and sealed under nitrogen flux to prevent oxygen contributions to the EPR signal at low temperatures. Measurements were performed in a commercial X-band (ν = 9.607GHz) EPR spectrometer (Bruker EMX-10) operation at a microwave power of 20 mW, with a 100 kHz modulation field of about 1.6 Oe (2 A m^−1^) in amplitude from 77 to 250 K and 4 to 300 K, respectively. Spectra double integration was obtained using WINEPR software (Bruker). The purified IGs spectra were fitted using Origin 7.5 with the HF. Gaussian and Lorentzian derivative-shaped lines were used to obtain the temperature dependence of the resonance linewidth (&delta;*H*_pp_), and resonance field (*H*_R_).

### IGs size measurements

A large layer of trophocytes and fat cells were freshly detached from the ventral abdomen in honeybee saline, mounted on the transfer slides with a little honeybee saline, covered with cover slides, and then the fluctuation of iron granules in size with or without 1 Gauss of magnetic field was measured under confocal laser scanning microscope (Leica TCS SP2 MP). The distribution of size was analyzed by photoshop software. The change rate (%) of IGs in size was calculated from the following equation:% change = the difference of size of IGs [(test-control)/control]×100%-the difference of size of white spot [(test-control)/control]×100%.

### Intracellular calcium measurements

Qualitative changes in [Ca^2+^]i levels were determined using the cell-permeable fluorescent [Ca^2+^]i indicator fluo-4-AM. A large layer of trophocytes and fat cells were freshly detached from the ventral abdomen in honeybee saline, loaded for 1 h with 5 µM fluo 4-AM at room temperature, centrifuged at 10,000 xg to wash fluo 4 out for 1 min twice, mounted on the transfer slides with a little honeybee saline, covered with cover slides, and then the variation of fluorescence intensity was measured under confocal laser scanning microscope (Leica TCS SP2 MP). The fluorescence intensity of trophocytes and fat cells were independently measured at the beginning time and at about 12 sec, respectively. 1 Gauss of magnetic field at the center of two magnets was applied to trophocytes and fat cells as closely as possible at about 12 sec, persisted for about 34 sec, and fluorescence intensity was measure at about 46 sec. Same experiments were done on controls without 1 Gauss of magnetic field. Fluo 4 was excited with the 488 nm line of a HeNe laser. The emitted light was collected at 505 nm [70].

Colchicine inhibits microtubule and latrunculin B inhibits microfilaments. A large layer of cells were freshly detached from the ventral abdomen in honeybee saline, treated with 1 µM colchicine and 62.5 nM latrunculin B for 30 min at room temperature, washed colchicine and latrunculin B out with honeybee saline for three times, loaded for 1 h with 5 µM fluo 4-AM at room temperature, centrifuged at 5,000 xg to wash fluo 4 out for 1 min twice, mounted on the transfer slides with a little honeybee saline, covered with cover slides, and the variation of fluorescence intensity was measured under confocal laser scanning microscope (Leica TCS SP2 MP). Fluorescence intensity of trophocytes and fat cells were independently measured at the beginning time and at about 12 sec, respectively. 1 Gauss of magnetic field at the center of two magnets was applied to trophocytes and fat cells as closely as possible at about 12 sec, persisted for about 34 sec, and fluorescence intensity was measured at about 46 sec. Same experiments were done on controls without 1 Gauss of magnetic field. Fluo 4 was excited with the 488 nm line of a HeNe laser. The emitted light was collected at 505 nm [70]–[73].
